# Genome-Wide Identification of the *SAMS* Gene Family in Upland Cotton (*Gossypium hirsutum* L.) and Expression Analysis in Drought Stress Treatments

**DOI:** 10.3390/genes13050860

**Published:** 2022-05-12

**Authors:** Fenglei Sun, Jun Ma, Penglong Wang, Yanlong Yang

**Affiliations:** 1 State Key Laboratory of Cotton Biology, Institute of Cotton Research of the Chinese Academy of Agricultural Sciences, Anyang 455000, China; 2 Research Institute of Economic Crops, Xinjiang Academy of Agricultural Sciences, Urumqi 830091, China; xj.majun@163.com; 3 Xinjiang Academy of Agricultural Sciences Kuqa County Upland Cotton Test Station, Xinjiang Academy of Agricultural Sciences, Kuqa 842000, China; 17591619607@163.com

**Keywords:** S-adenosylmethionine synthase, synteny, drought resistance, qRT-PCR, cotton

## Abstract

Cotton is an important commercial crop whose growth and yield are severely affected by drought. S-adenosylmethionine (SAM) is widely involved in the plant stress response and growth regulation; however, the role of the S-adenosylmethionine synthase (*SAMS*) gene family in this process is poorly understood. Here, we systematically analyzed the expression of *SAMS* genes in Upland Cotton (*Gossypium hirsutum* L.). A total of 16 *SAMS* genes were identified, each with a similar predicted structure. A large number of cis-acting elements involved in the response to abiotic stress were predicted based on promoter analysis, indicating a likely important role in abiotic stress responses. The results of qRT-PCR validation showed that *GhSAMS* genes had different expression patterns after drought stress and in response to drought stress. Analysis of a selected subset of *GhSAMS* genes showed increased expression in cultivar Xinluzhong 39 (drought resistant) when compared to cultivar Xinluzao 26 (drought-sensitive) upland cotton. This study provides important relevant information for further study of *SAMS* genes in drought resistance research of upland cotton, which is helpful for drought-resistance improvement of upland cotton.

## 1. Introduction

When crops are subjected to drought stress, yield is reduced, and crop growth, development and metabolism are severely affected [1,2]. During the long-term evolution of crops, related defense mechanisms and metabolites were formed to cope with drought stress [3]. S-adenosylmethionine synthase is an important protein involved in plant stress response and is the only enzyme that catalyzes the synthesis of S-adenosylmethionine (SAM) [4]. SAM enzymes are widely involved in plant stress response, growth and development regulation [5,6]. Studies have reported that SAM is involved in methylation reactions, which mainly regulate gene expression and maintain genome functions [7]. In recent years, researchers have excavated and identified many *SAMS* genes [8,9]. There are four *SAMS* genes in *Arabidopsis thaliana*, among which, *AtSAMS3* is mainly expressed in pollen [8,9]. In rice, there are three *SAMS* genes [10]. Related studies have shown that *SAMS* is involved in plant abiotic stress response. In tomato, Espartero et al. found that *SAMS* was differentially expressed in tomato after salt stress [11]. In cucumber, *SAMS* was induced to express after salt stress and participated in related regulation [12,13]. Interestingly, soybean *SAMS* was differentially expressed under drought and waterlogging stress, but not sensitive to NaCl and low-temperature treatments [14,15].

At present, upland cotton gene sequencing has been completed. With the development of sequencing technology, genome-wide analysis data provide us with important information on cotton response to drought stress when conducting related gene family analysis, and provide us with a theoretical basis for related research [16].

Although *SAMS* genes have been reported in crops such as Arabidopsis, rice and tomato, there are few related studies on the *SAMS* gene family in cotton, especially the role of *SAMS* genes in drought tolerance [10,17]. Therefore, identification of drought-resistance-related genes is helpful for drought-resistance improvement and breeding of upland cotton, and can provide candidate genes related to drought resistance. This also has important implications for the study of *SAMS* genes. For the first time, we performed the whole gene identification and analysis of the *SAMS* gene family in upland cotton. In this study, a total of 16 *SAMS* genes were identified in upland cotton, and the phylogenetic analysis, gene structure and chromosome distribution analysis of the upland cotton *SAMS* gene family were completed. In addition, the gene expression data of the *GhSAMS* gene in different tissues from the cotton database were extracted, and the expression patterns in different tissues were analyzed. At the same time, the response of the *GhSAMS* gene to drought stress in materials with different drought resistance was analyzed. In this study, the *GhSAMS* gene family was systematically analyzed, and the expression pattern under drought stress was analyzed to provide a relevant theoretical basis for future research on the cotton *SAMS* gene family.

## 2. Materials and Methods

### 2.1. Sequence Identification of SAMS Proteins

The upland cotton (*Gossypium hirsutum* L., ZJU v2.0) genome sequence for this study was downloaded from CottonFGD (http://cottonfgd.org/ accessed on 8 April 2022) [18]. The genomic data of Arabidopsis thaliana (*Arabidopsis thaliana* L.) were downloaded from the Phytozome v12 database (http://phytozome.jgi.doe.gov/pz/portal.html accessed on 8 April 2022) [19].

The Hidden Markov Model (HMM) (PF02772, PF02773, PF00438) of S-adenosylmethionine synthase with the keyword “S-adenosylmethionine synthase” was downloaded from the Pfam database (https://pfam.xfam.org/ accessed on 8 April 2022) [20], and the S-adenosylmethionine synthase protein sequence was obtained by BLAST alignment in the local protein database by HMMER3.0 software (e-value 10^−5^) (//hmmer.org) [21]. The conserved domain of “S-adenosylmethionine synthase” was further examined by Pfam 32.0 (http://pfam.xfam.org/ accessed on 8 April 2022) and NCBI-CDD (http://www.ncbi.nlm.nih.gov/cdd accessed on 8 April 2022) [22], and other redundant sequences were manually removed. Physicochemical properties of SAMS proteins were calculated and subcellular localization predictions obtained using ProtParam (https://web.expasy.org/protparam/ accessed on 8 April 2022) [23] and CELLO RESULTS (http://cello.life.nctu.edu.tw/ accessed on 8 April 2022) online software [24]. In addition, the hydrophilicity (GRAVY) of SAMS protein was analyzed using ProtParam online tool (http://web.expasy.org/protparam accessed on 8 April 2022). Selected *SAMS* genes were named by their chromosomal location (bp) on the At subgenomic chromosome and the Dt subgenomic chromosome.

### 2.2. Analysis of Phylogenetic, Gene Structure and Conserved Motif Characteristics

The sequences of SAMS proteins of three plant species, cotton, Arabidopsis and rice, were analyzed using MEGA7.0 [25], and ClustalX [26] was used for multiple sequence alignment after importing the sequences. Neighbor-joining (NJ) methods and maximum similarity (ML) were used in the analysis, both with 1000 bootstrap replicates [27,28]. The exon–intron structure of the *SAMS* genes was analyzed using GSDS 2.0 (http://gsds.cbi.pku.edu.cn/ accessed on 8 April 2022) [29]. Conserved domains of SAMS proteins were analyzed by MEME (http://meme.sdsc.edu/meme/cgi-bin/meme.cgi accessed on 8 April 2022) [30].

### 2.3. Chromosomal Location, Collinearity and Promoter Analysis

Chromosome-specific positions of *SAMS* genes were obtained by setting default search criteria in the Phytozome database and cottonFGD database. The upland cotton *SAMS* genes showed different specific distributions on the chromosomes of the At subgenome and the Dt subgenome, and then according to their specific physical locations (bp), the distribution map of the SAMS genes on the upland cotton chromosomes was drawn in the TBtools software. [31]. Collinearity of homologous genes of SAMS proteins in upland cotton and Arabidopsis was analyzed by MCScanX (http://chibba.pgml.uga.edu/mcscan2/ accessed on 8 April 2022) [32], and gene duplication events between the two were visualized using TBtools. The 2000 bp promoter sequence of the upland cotton *SAMS* genes was downloaded from cottonFGD database. PLACE (http://www.dna.affrc.go.jp/PLACE accessed on 8 April 2022) was used to analyze the promoter sequences of upland cotton *SAMS* genes [33].

### 2.4. Digital Expression Analysis of SAMs Genes in Cotton

The FPKM (fragments per kilobase per million reads) values for the transcriptome data of six tissues (Sepal, Leaf, Pistil, Root, Stem, and Torus) of the upland cotton *SAMS* gene were downloaded from the cottonFGD database. At the same time, the gene expression data of *GhSAMS* gene under stress such as drought and salinity were obtained. The obtained data were finally visualized and the heat map was mapped by the TBtools software.

### 2.5. Drought Treatment and PCR (qRT-PCR) Analysis of Cotton Drought Resistant Materials

After sterilizing upland cotton seeds (Xinluzhong 39 (drought resistant) and Xinluzao 26 (drought-sensitive) with 75% ethanol for 5 min, the surfaces were re-sterilized with 0.5% sodium hypochlorite (NaClO), and then the surfaces were rinsed again with sterile water. [32,34,35]. The seeds were then placed in a Petri dish lined with moistened filter paper and transferred to an incubator until germination. Seedlings of uniform size were then grown in sterile soil to the three true leaf stages at 25 °C and a 16/8 h (light/dark) photoperiod [32,35]. The soil drought stress test treatment was carried out at the stage of growing to three true leaves, and cotton roots, stems and leaves were collected at 0, 1, 3, 6, 12 and 24 h, respectively (the stress treatment lasted 24 h). the collected sample material was stored in an ultra-low temperature freezer at −80 °C.

The total RNA of the drought stress experimental samples was extracted using an RNA kit (TIANGEN Biotech, Beijing, China), and the first-strand cDNA was synthesized by reverse transcription. The final PCR amplification product was verified by 2% agarose gel electrophoresis. The qRT-PCR assay was performed in ABI Prism7500, the internal reference control gene was UBQ7, and each sample was replicated 3 times (including biological replicates). Finally, the 2^−ΔΔCt^ method was used to analyze the expression of cotton *SAMS* genes [36].

### 2.6. Protein Interaction Network Analysis of GhSAMS

Through protein homology analysis in the Arabidopsis information resource database (https://www.arabidopsis.org/ accessed on 8 April 2022), the homologous genes of GhSAMS were screened out and submitted to the STRING (https://string-db.org/ accessed on 8 April 2022) database together. The relationship between the Arabidopsis protein–protein interaction network and the cotton protein–protein interaction network was obtained through the STRING database, respectively. Finally, through the comparison of Arabidopsis interaction genes, the homologous genes of upland cotton were screened, the interaction network of GhSAMS was analyzed in combination with the interaction genes of upland cotton, and the Cytoscape-3.8.2 software was used for visualization [37,38].

## 3. Results

### 3.1. Identification of SAMS Genes in Upland Cotton

Different transcripts and redundant sequences of the same gene were manually checked and deleted by screening the B-box domain. Ultimately, a total of 16 SAMS sequences were identified in upland cotton (Table 1). A sequential numbering was performed according to the order of the *GhSAMS* genes in the chromosomal location (Table 1).

The physicochemical properties of all the upland cotton SAMS proteins were further analyzed, and the results showed that all the upland cotton SAMS proteins were very different in terms of protein length, protein molecular weight (MW) and isoelectric point (pI). Upland cotton SAMS proteins have an average length of 383 amino acids and vary in length from 256 (*GhSAMS3*) to 393 (*GhSAMS1*) amino acids (Table 1). The isoelectric point (pI) values and molecular weights of the upland cotton SAMS protein sequences ranged from 5.48 to 8.73 and 28,120.07 to 43,090.84 Da, respectively. Subcellular localization prediction results showed that the localization predictions of 16 SAMS proteins were all displayed in the cytoplasm. The overall mean of the hydrophilicity (GRAVY) scores for all SAMS proteins was negative, indicating that SAMS proteins are hydrophilic and likely localized in the cytosol, which is consistent with predictions of subcellular localization (Table 1).

### 3.2. Phylogenetic, Gene Structure and Motif Identification of Upland Cotton SAMS Genes

**Figure 1:**
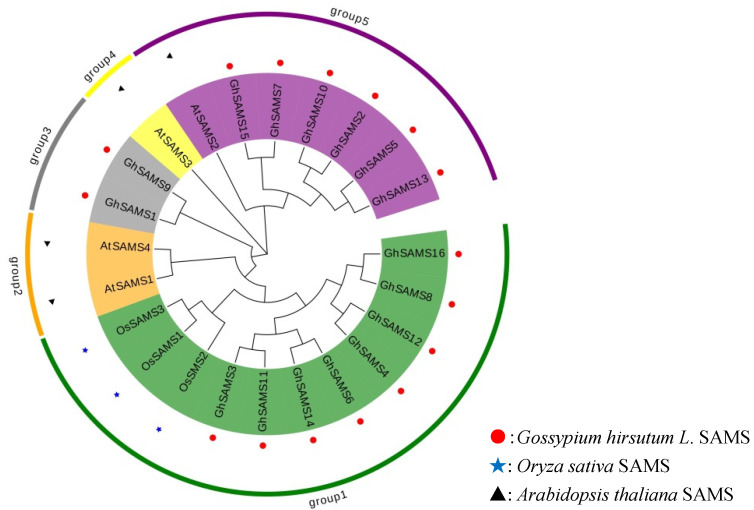
Phylogenetic analysis of *SAMS* genes in cotton, Arabidopsis and *Oryza sativa*.

To analyze the evolutionary relationship of *SAMS* genes, we performed a phylogenetic analysis of the conserved sequences of 21 different SAMS proteins. These include 16 upland cotton SAMS sequences, 3 rice SAMS sequences and 5 Arabidopsis sequences. The phylogenetic tree in this study divided SAMS proteins into five categories and the results are shown in Figure 1. The first group includes 11 members, including 8 members of cotton and 3 members of rice; the second group includes 2 members, mainly Arabidopsis *thaliana* sequences; the third group includes 2 members of cotton; the fourth group includes 1 member of Arabidopsis; and the fifth group includes 6 members of cotton and 1 member of Arabidopsis (Figure 1).

**Figure 2:**
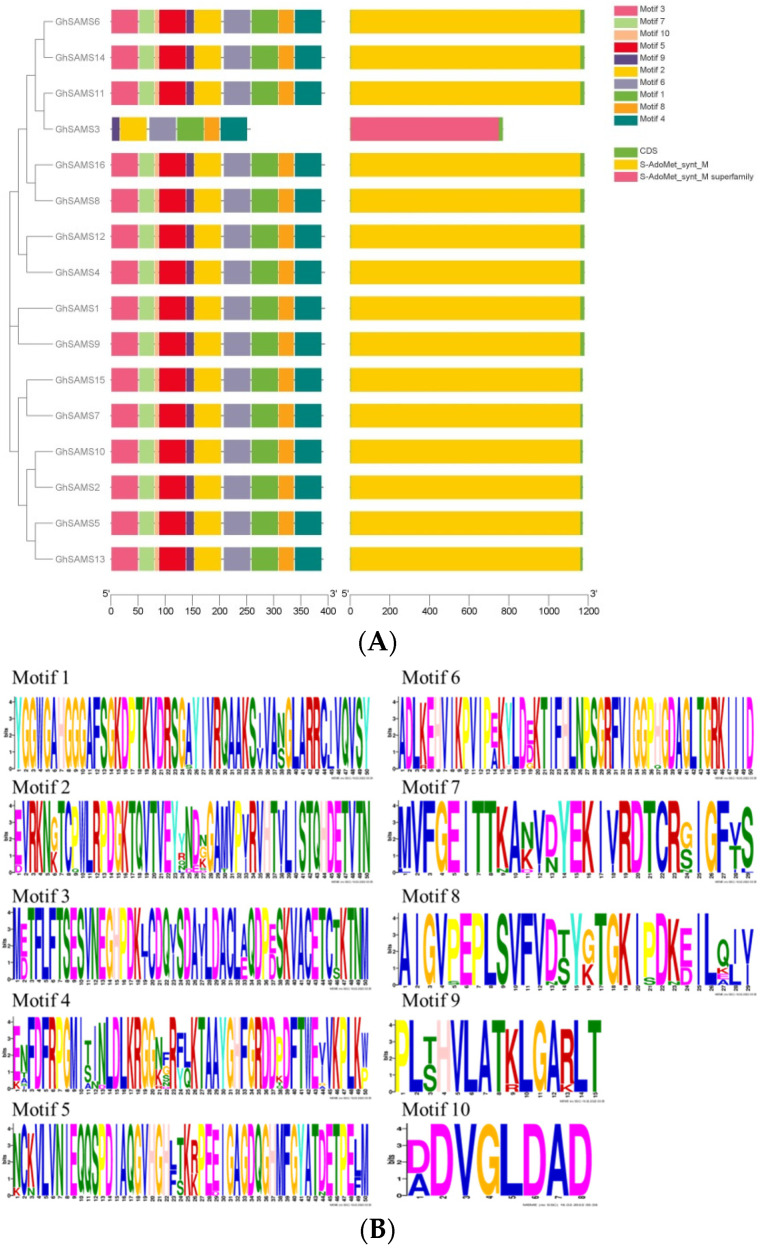
Sequence conservation analysis of SAMS proteins: (**A**) Alignment of the conserved domains of SAMS proteins and genomic exon–intron structures in upland cotton. (**B**) Ten conserved motifs.

Analysis of the cotton *SAMS* gene structure reveals a rare distribution of exonic regions; the key evolutionary changes in *SAMS* genes in the cotton genome are shown. Among them, the shortest gene among all *SAMS* genes is *GhSAMS3* with a length of only 771 bp, while the longest identified is *GhSAMS1* with a genome sequence of 1182 bp (Table 1). The clustering among all genes of cotton *SAMS* showed a highly similar gene structure, and the gene structure was almost the same with little difference (Figure 2A).

The S-adenosylmethionine synthase domain is about 300 amino acid residues long and is considered a key element. Motifs of SAMS were predicted by the MEME program, and ten different motifs were identified based on the alignment of conserved domains in *GhSAMS* (Figure 2) where the conservation of SAMS protein sequence positions is represented by the cumulative height of all letters, and the frequency of amino acids is represented by the height of the letters. The details of the motifs are listed in Table S1. The predicted structure of SAMS proteins can be divided into two categories through the results of prediction analysis, which are different in upland cotton. It contains two types, namely, *GhSAMS3* is an S-AdoMet_synt_M superfamily, and the rest are AdoMet_synt_M types (Figure 2A). The distributed and relative positions of motifs in conserved regions of *GhSAMS* proteins are relatively consistent and may be decisive for the development of specific phenotypes.

### 3.3. Chromosomal Location, Collinearity and Promoter Analysis of the SAMS Genes of Upland Cotton

**Figure 3:**
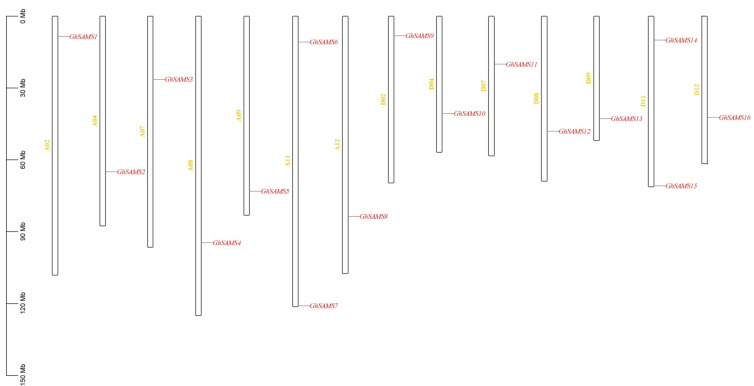
Chromosome distribution of upland cotton *SAMS* genes.

Mapping cotton *SAMS* genes to their chromosomes, the results showed that 16 *SAMS* genes were evenly distributed on 14 chromosomes. Meanwhile, the exact position (in bp) of each cotton *SAMS* gene on the cotton chromosome is given in Table 1. Each chromosome contains only one gene (Figure 3), and the location of each cotton *SAMS* gene also varies. Interestingly, the mapping results showed that the numbers of *GhSAMS* genes in the At subgenome and Dt subgenome of upland cotton were basically the same; this shows that there is no obvious preference for the retention and loss of *GhSAMS* genes on homologous chromosomes between subgenomes. On different chromosomes, most of the *GhSAMS* genes are located at opposite ends of the chromosome (upper or lower end). (Figure 3). However, the *GhSAMS* genes were evenly distributed on the two homologous chromosomes in the two subgenomes of the tetraploid upland cotton, but the distribution on chromosomes was uneven, and uneven distribution is independent of chromosome length. (Figure 3).

**Figure 4:**
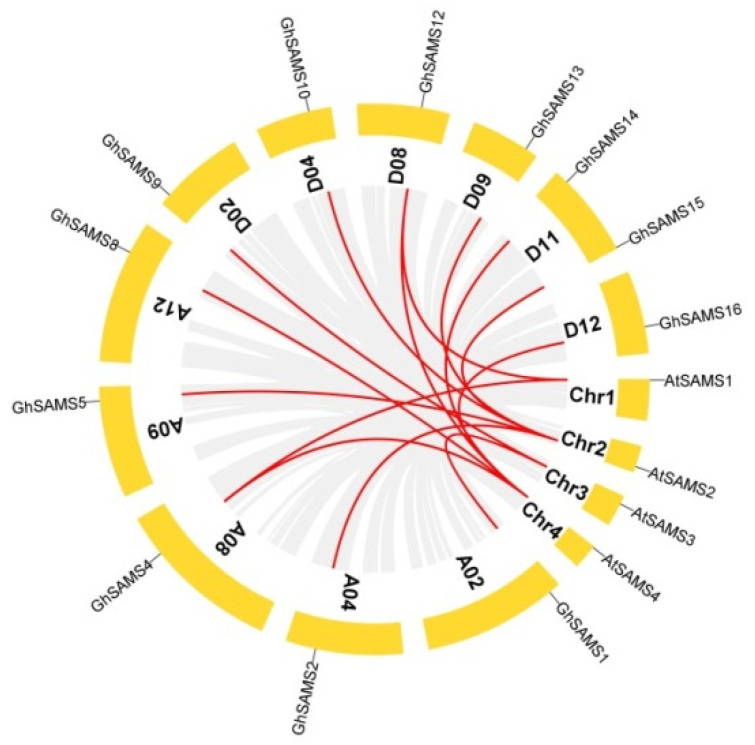
Collinearity analysis and comparison of the *SAMS* genes between upland cotton) and Arabidopsis.

To fully understand the collinearity between cotton and Arabidopsis *SAMS* gene families, combinatorial analysis of cotton and Arabidopsis *SAMS* genes was performed. The results of collinearity analysis showed that there were 14 (87.5%) collinear gene pairs in the upland cotton and Arabidopsis genomes. We draw a collinearity comparison plot between cotton and Arabidopsis and listed the details of the homologous gene pairs in Table S2. There were 14 pairs of homologous *SAMS* genes between upland cotton and Arabidopsis, of which 6 pairs were shown between the A genome of upland cotton and the Arabidopsis genome, and 8 pairs were shown between the D genome and the Arabidopsis genome (Figure 4). The results of the collinearity analysis revealed that the main expansion mechanism of the *SAMS* gene family is WGD/segmented and scattered duplication events, rather than proximal and tandem duplication events.

**Figure 5:**
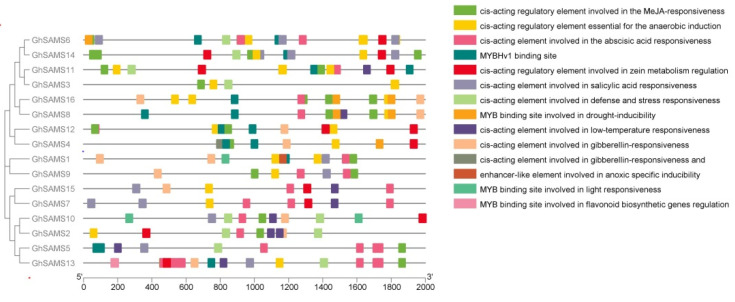
Predictive analysis of cis-acting regulatory elements in the promoter region of *GhSAMS*.

Analysis of cis-acting elements in the *SAMS* genes promoter region using PLACE can predict the relevant functions of the *SAMS* genes in upland cotton. Fourteen cis-acting elements were mainly included in the predicted results of PLACE analysis (Figure 5). Three genes detected MYB binding sites in the promoter regions and were involved in the regulation of flavonoid biosynthesis-related genes. MYB binding sites related to drought induction were detected in the promoter region of eight genes. In addition, an enhancer-like element involved in anoxic specific inducibility was identified in the promoters of 28 genes. At the same time, cis-acting elements involved in defense and stress response and enhancer-like elements related to hypoxia-specific induction were also identified in the promoter regions of 12 genes. These data may suggest that the upland cotton *SAMS* genes play important roles in response to various abiotic stresses. (Figure 5).

### 3.4. Expression Analysis of SAMS Genes in Upland Cotton

**Figure 6:**
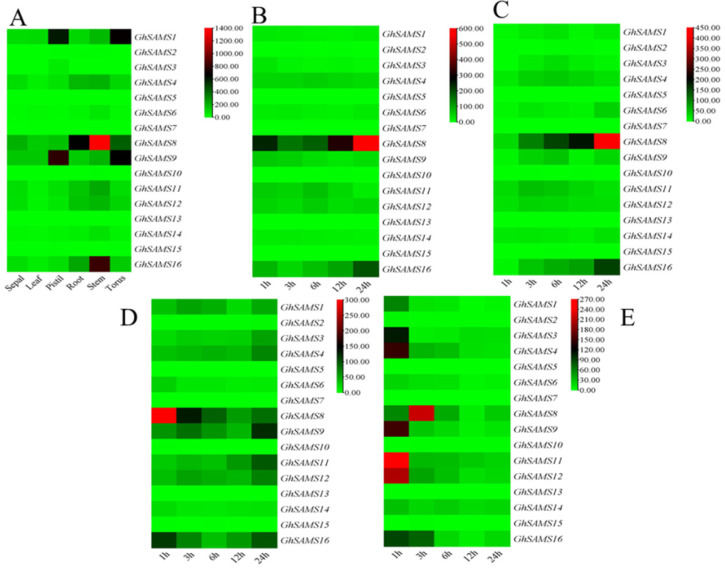
(**A**) Expression analysis of *GhSAMS* gene (log_2_(FPKM)). (**B**) Expression analysis of *GhSAMS* gene after drought stress. (**C**) Expression analysis of *GhSAMS* gene after salt stress. (**D**) Changes in *GhSAMS* gene expression in different periods after cold stress. (**E**) Changes in *GhSAMS* gene expression in different time periods after heat stress.

The expression levels of *SAMS* genes in multiple tissue types were analyzed through previously published RNA-seq data to analyze the critical role of *SAMS* genes in organ development in upland cotton. According to the heat map, we found that *GhSAMS1* and *GhSAMS9* were specifically expressed in pistils and receptacles, while *GhSAMS8* and *GhSAMS16* were mainly expressed in stems and roots, and to a lesser extent in other tissues. *GhSAMS4*, *GhSAMS11* and *GhSAMS12* were also specifically expressed in stems and roots (Figure 6).

The expression levels of *SAMS* genes under abiotic stresses such as drought, salt, heat and cold were further analyzed by RNA-seq data. The results of heat map analysis showed that *GhSAMS8* and *GhSAMS16* were significantly up-regulated at 24 h of stress (Figure 6). However, *GhSAMS4*, *GhSAMS11* and *GhSAMS12* were significantly up-regulated at 6 h of stress. The expression under salt stress was basically the same as that under drought stress. Under heat stress, *GhSAMS8* and *GhSAMS16* were significantly up-regulated at 1 h, while the expression levels of *GhSAMS4*, *GhSAMS9*, *GhSAMS11* and *GhSAMS12* were significantly higher at 24 h. Under low-temperature stress, all the above genes except *GhSAMS8* were significantly up-regulated at 1 h of stress, while *GhSAMS8* was significantly up-regulated at 3 h of stress (Figure 6).

### 3.5. Expression Analysis of GhSAMS after Drought Stress

**Figure 7:**
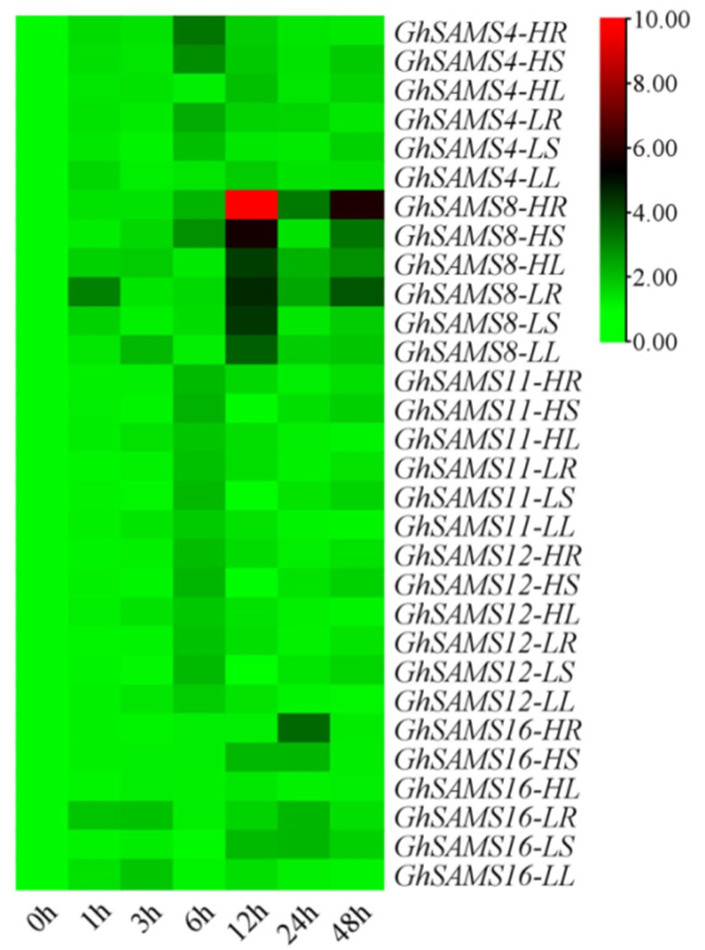
The expression of *GhSAMS* under drought stresses (log_2_(FPKM)).

Information about gene function can be provided by analysis of the expression level of the gene. To analyze the role of *GhSAMS* genes in abiotic stress response, from the results obtained from the RNA-seq data analyzed in the previous section, five genes were selected because they may play a role in drought stress response. The expression levels of the five candidate genes were analyzed by qRT-PCR after 1 h, 3 h, 6 h, 12 h, 24 h and 48 h after drought stress in Xinluzhong 39 and Xinluzao 26 upland cotton varieties. Gene-specific primers are listed in Table S3. The results of the qRT-PCR analysis showed that these five genes responded to drought stress through changes in their expression patterns during the drought stress in Xinluzhong 39 and Xinluzao 26. Xinluzao 26 and Xinluzhong 39 showed sensitivity and resistance to drought stress, respectively, after being subjected to drought stress. The results showed that *GhSAMS* genes were induced at different time points after drought stress and rapidly reached the peak expression level. The results of the heat map analysis showed that the five *GhSAMS* genes all showed different degrees of up-regulation (>2-fold) and expression after drought stress. The results of the heat map analysis showed that the five *GhSAMS* genes were up-regulated (>2-fold) to varying degrees in both drought-sensitive cultivar Xinluzao 26 and drought-resistant cultivar Xinluzhong 39 after being subjected to drought stress. As shown in Figure 7, especially when drought stress was for 12 h, the expression of the *GhSAMS8* gene was up-regulated (>2-fold) in drought-resistant cultivar Xinluzhong 39, and was significantly higher than that in drought-sensitive cultivar Xinluzao 26. *GhSAMS4*, *GhSAMS11* and *GhSAMS12* showed the highest expression levels in drought-resistant cultivar Xinluzhong 39 at 6 h, and were more than 1-fold higher than drought-sensitive material Xinluzao 26 at 6 h (Figure 7). The overall expression level of *GhSAMS16* was the highest at 24 h, which was more than onefold higher than that of Xinluzao 26 (Figure 7). Comparative analysis of *GhSAMS* genes expression patterns at six time points after drought stress treatment showed that the expression levels of five genes in cultivar Xinluzhong 39 were higher than those in cultivar Xinluzao 26 (Figure 7). These results suggest that all five selected genes are induced by drought stress, even though their expression levels differed after stress.

### 3.6. GhSAMS Protein Interaction Network Analysis

**Figure 8:**
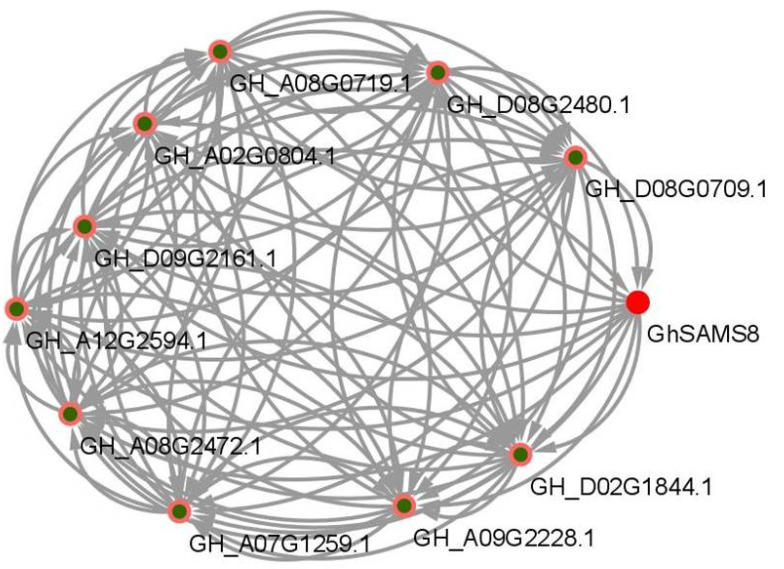
Interaction network of GhSAMS8 proteins in upland cotton.

Most proteins cannot directly participate in the stress response of plants alone, and many physiological processes in plants are completed through protein–protein interactions [39]. In order to analyze and understand the molecular mechanism of drought tolerance of the *GhSAMS8* gene, the interaction network between the *GhSAMS8* gene protein and other upland cotton proteins was constructed based on the Arabidopsis homologous gene and its own interaction network (Figure 8). The analysis results showed that the GhSAMS8 protein interacts with 10 upland cotton proteins. Only GH_D08G0709.1, GH_D08G2480.1 and GH_D09G2161.1 were located upstream of GhSAMS8, suggesting that GH_D08G0709.1, GH_D08G2480.1 and GH_D09G2161.1 may interact with the GhSAMS8 domain. More genes were bound to the downstream S-AdoMet_synt_M domain of GhSAMS8, which further indicated the complex function of the *SAMS* gene family and the potential role of *GhSAMS8* in response to drought stress in upland cotton (Figure 8).

## 4. Discussion

In this study, we identified 16 *SAMS* genes in upland cotton, compared with 5 and 3 reported in Arabidopsis and rice, respectively [40]. The involvement of *SAMS* genes in various metabolic pathways is related to stress resistance [9,41]. The number of *GhSAMS* genes on chromosomes is relatively small, and their distribution on chromosomes presents a certain physical location, while the number and physical regions of *GhSAMS* genes on chromosomes do not affect gene expression [42]. Previous reports have indicated that the distribution pattern of introns/exons in genes plays an important role in related biological functions [43]. Exon–intron loss occurs in the chromosomal rearrangement or fusion of gene families, which affects the evolution of exon–intron diversity in this process [44,45]. In this study, the exon numbers of all 16 *GhSAMS* genes were basically the same, and the structures of all exons were basically similar. Similar exon numbers and structures were also shown in the phylogenetic tree. In this study, the similarity of the gene structure makes the prediction results of gene motifs basically similar. Although the gene structures are similar, the encoded amino acids are different, which leads to different catalytic activities and functions.

Whole-genome duplication (WGD) events affected the expansion of the *SAMS* gene family in upland cotton [45,46]. In the process of plant evolution, the polyploidy of plants will cause many repetitive genes to appear in the plant genome, which results in many changes in gene expression and genome in plant tissues. [23,47,48], and these specific gene-encoding biotic and abiotic exogenous factors retain their associated structures and functions after replication [23,45]. In this study, the results of *GhSAMS* genes’ phylogenetic evolution, gene structure and collinearity analysis showed that the homology and gene structure of *GhSAMS* genes were basically consistent with the above viewpoints (Figure 1 and Figure 5). In addition, the results of the evolutionary analysis show that the *GhSAMS* gene family is very strict and conserved during its species expansion, which may be related to its need to maintain related functions. For example, the *SAMS* gene of Arabidopsis was down-regulated under abiotic stress conditions such as salt, heat and temperature stress [39]. The presence or absence of related cis-elements in the promoter of a gene has an important impact on the related functions of the gene. For example, in Arabidopsis, dehydration response elements (DREs) bind to associated transcription factors to regulate drought- and heat-responsive genes [49,50]. At present, MYB is an important transcription factor related to plant drought resistance and plays an important role in the transcriptional regulatory network of plant drought resistance [51,52]. Here, the results of our promoter prediction analysis revealed that five *GhSAMS* genes were upregulated after drought stress, and their promoter regions contained MYB-responsive cis-acting regulatory elements (Figure 5).

The tissue expression pattern analysis of RNA-seq data provides an important theoretical basis for the functional analysis of *GhSAMS* genes after drought stress. According to the results of qRT-PCR analysis, these five genes were significantly up-regulated (>2-fold) in Xinluzhong 39 and Xinluzao 26 materials (Figure 7). These five genes were significantly induced and expressed by drought stress treatment, and initially mainly contributed to the drought stress response. The results of differential expression analysis of these five genes showed that the *GhSAMS* genes were highly expressed in the roots of cotton under drought stress, which mainly affected the physiological process of the roots and improved their tolerance to drought stress. High expression of these genes in roots helped alleviate drought stress (Figure 7).

## 5. Conclusions

In this study, 16 *SAMS* genes were identified in upland cotton by genome-wide analysis, and these *GhSAMS* genes were divided into five main groups, in total, by phylogenetic analysis. A large number of abiotic stress-related cis-acting elements in promoters were predicted to show their role in abiotic stress tolerance. The expression pattern of *GhSAMS* genes after drought stress was analyzed by qRT-PCR, and the results showed that *GhSAMS* gene expression was induced by drought stress. The analysis of the cotton *SAMS* gene family will help us understand the role of *SAMS* genes in drought stress and to provide a theoretical basis for further analysis of the function of the *GhSAMS* gene family and its potential in the genetic improvement of cotton drought resistance.

## Figures and Tables

**Table 1:** A catalog of 16 cotton SAMS proteins.

NIPGR ID.	Cotton Identifier	Chromosome	Start Position	End Position	Protein (aa)	pI	Molecular Weight (Da)	CDS (bp)	Predicted Subcellular Location	GRAVY
*GhSAMS1*	GH_A02G0616.1	AD1-A02	8,514,170	8,515,351	393	5.680	43,060.86	1182	Cytoplasmic	−0.308
*GhSAMS2*	GH_A04G0904.1	AD1-A04	65,039,231	65,040,403	390	6.510	42,854.92	1173	Cytoplasmic	−0.326
*GhSAMS3*	GH_A07G1419.1	AD1-A07	26,342,235	26,343,005	256	8.730	28,120.07	771	Cytoplasmic	−0.326
*GhSAMS4*	GH_A08G1451.1	AD1-A08	94,661,115	94,662,296	393	5.590	43,090.84	1182	Cytoplasmic	−0.325
*GhSAMS5*	GH_A09G1633.1	AD1-A09	73,132,352	73,133,524	390	6.640	42,609.68	1173	Cytoplasmic	−0.299
*GhSAMS6*	GH_A11G1106.1	AD1-A11	10,728,104	10,729,285	393	5.480	43,025.52	1182	Cytoplasmic	−0.360
*GhSAMS7*	GH_A11G3695.1	AD1-A11	120,911,380	120,912,552	390	6.320	42,681.58	1173	Cytoplasmic	−0.332
*GhSAMS8*	GH_A12G1381.1	AD1-A12	83,660,094	83,661,275	393	5.490	43,070.68	1182	Cytoplasmic	−0.335
*GhSAMS9*	GH_D02G0631.1	AD1-D01	8,142,070	8,143,251	393	5.650	43,043.83	1182	Cytoplasmic	−0.300
*GhSAMS10*	GH_D04G1227.1	AD1-D04	40,645,773	40,646,945	390	6.500	42,811.94	1173	Cytoplasmic	−0.306
*GhSAMS11*	GH_D07G1411.1	AD1-D07	20,066,986	20,068,167	393	5.490	43,038.68	1182	Cytoplasmic	−0.328
*GhSAMS12*	GH_D08G1473.1	AD1-D08	48,053,425	48,054,606	393	5.520	43,039.75	1182	Cytoplasmic	−0.338
*GhSAMS13*	GH_D09G1576.1	AD1-D09	42,758,795	42,759,967	390	6.640	42,694.79	1173	Cytoplasmic	−0.294
*GhSAMS14*	GH_D11G1137.1	AD1-D11	9,955,464	9,956,645	393	5.490	43,061.63	1182	Cytoplasmic	−0.358
*GhSAMS15*	GH_D11G3723.1	AD1-D11	70,868,476	70,869,648	390	6.320	42,615.56	1173	Cytoplasmic	−0.320
*GhSAMS16*	GH_D12G1397.1	AD1-D12	42,290,014	42,291,195	393	5.490	43,041.68	1182	Cytoplasmic	−0.321

## Supplementary Materials

The following supporting information can be downloaded at: https://www.mdpi.com/article/10.3390/genes13050860/s1, Table S1. Multilevel consensus sequences of motifs in GhSAMS, Table S2: Orthologous and paralogous SAMS gene pairs among upland cotton, and Arabidopsis. Table S3: Primers used in this study.
